# The Death of Sir Edward Wood: A Notable Hospital Chairman

**Published:** 1917-09-29

**Authors:** 


### THE DEATH OF SIR EDWARD WOOD.

### A Notable Hospital Chairman.

By the death of Sir Edward Wood, who was so prominently associated with the great improvement and reconstruction scheme of the Leicester Royal Infirmary, there passes from our midst one of the best known of modern hospital chairmen. In the issue of The Hospital of November 5, 1910, we dealt at length with Sir Edward Wood's great work for the Leicester Royal Infirmary, and outlined the circumstances in which he was induced, much against his own inclination, to accept the chairmanship of the institution at a time when the mayoralty of the borough was occupying much of his work and attention.

### The Chairman's First Work.

Once having accepted the position, Sir Edward Wood found himself confronted with the task of having to raise a large sum of money to bring the institution up to modern standards. Fifty thousand pounds was required as a commencement. It seemed an impossible sum to ask for, but Sir Edward was possessed of enthusiasm, and estimated at its true worth the force of personal example. With a contribution of £5,000 from himself, a similar sum from his firm, and other contributions, he at once announced that a quarter of the sum required was promised. This magnificent start appealed to Leicester's business men, and the following improvements were thereupon undertaken:—(1) The reconstruction of the entire drainage system; (2) the enlargement of the ground area by the purchase of property in Parliament and Knighton Streets at a cost of £10,000; (3) the erection of a new boiler-house, steam disinfector, and incinerator; (4) the reconstruction of the heating system by the installation of modern boilers and a modern pump-room; (5) the reconstruction of six of the oldest wards and the addition of separate sanitary towers; (6) the reconstruction and refitting of the kitchens; (7) the erection of a new out-patient department.

### A Second £50,000 for Further Reconstruction.

This large scheme completed, Sir Edward Wood found himself up against new needs which could not be turned down—viz., the additional bed accommodation, and consequentially the erection of an adequate nurses' home.

The new scheme was estimated to cost another £50,000, and Sir Edward boldly and confidently entered upon the task of raising the money. An additional contribution of £5,000 from himself was the first indication to the governors of the infirmary that Sir Edward Wood was "out to succeed," and when a few days later he announced that the family of his old friend Mr. George Oliver were desirous of contributing £6,000 to name a ward in the projected new wing, authority was readily given to proceed with the building, which was to contain ninety-nine beds. Before it was completed Sir Edward Wood's donation list showed that his friends had so loyally supported his appeal that £5,000 was in hand, after providing for the additional accommodation for patients, for the projected nurses' home. Thus, including a donation of £6,000 from Mr. Samuel Odames, in commemoration of his eighty-fourth birthday, for naming the "Samuel Odames" Ward, practically half of the cost (£24,000) of the new nurses' home, containing ninety-eight bedrooms and adequate sitting-room, recreation, and reading room accommodation, was provided, and the foundation-stone was laid on November 4, 1908, by Miss Gertrude Rogers, the lady superintendent.

A full description of the building appeared in The Hospital of February 5, 1910. On February 8, 1910, the building was opened, and was formally and appropriately designated "The Edward Wood Home," in recognition of the chairman's personal generosity and great services.

### The New Children's Hospital.

After the expenditure of a further £3,000 on laundry buildings and machinery for this department Sir Edward took up arms on behalf of the children. For some time past the children's hospital had been unsatisfactory, owing to outbreaks of infectious disease, the cause of which could not be ascertained with certainty. Sir Edward soon convinced the board that no policy save that of entire reconstruction would be efficient. Plans were submitted by the architect for modernising the children's hospital at a cost of £8,000, and were approved. The work was soon afterwards taken in hand, but as it proceeded it was plainly evident that additional improvements could be incorporated. These were carried out as they presented themselves, and brought the cost of the reconstruction to £14,800, the last £2,500 of which was provided a few months after the opening.

### Ill-Health and Retirement.

In December 1914, as his health seemed to be completely broken, Sir Edward Wood asked the governors to allow him to resign the chairmanship of the hospital, which he had occupied since 1902. Glowing tributes were paid by the board and the honorary medical staff to his great abilities as a hospital chairman and administrator, and deep regret was expressed at his retirement.

A partial recovery to health and one or two short visits to the scene of his former labours induced the hope that the infirmary might enjoy his presence as a member of the board, but these hopes were doomed to failure. Sir Edward became a permanent invalid, and passed to the vale beyond on September 22, in the seventy-ninth year of his age.

The words of Colonel C. J. Bond at the meeting of the governors when Sir Edward Wood's resignation was accepted are particularly appropriate because of their wide and general application:

"Two feelings were uppermost in their minds, one the feeling of regret at losing a man of Sir Edward Wood's calibre, and the other, a feeling of thankful-

(Continued on page 530.)

### THE DEATH OF SIR EDWARD WOOD—(*continued from p. 531*).

ness that it had been given to a Leicester citizen to accomplish the work which Sir Edward had done for that institution during the last thirteen years. That work would stand out in hospital administration and hospital enterprise even if they took the country over. Sir Edward had raised the standard of public service, and he had also raised the standard of giving in Leicester. He had given the best of himself, and he had sacrificed personal interest and leisure which after all were the true tests of service to the community."

No greater instance of his intense love for the institution can be recorded than the offer of £1,000 to his successor in the chairmanship of the Leicester Royal Infirmary to start a fund for the building of a new pathological department, an offer which enabled the entire cost of the department to be subsequently raised.

Sir Edward Wood was thrice Mayor of the Borough, and leaves three married daughters to mourn his loss, a loss which is shared by every resident in the Borough.

